# Acknowledgements to Referees

**DOI:** 10.1186/s10194-015-0499-3

**Published:** 2015-02-27

**Authors:** Valerio De Angelis

**Affiliations:** Assistant to the Editor, The Journal of Headache and Pain, Via di Grottarossa 1035, 00189 Rome, Italy

## Abstract

The quality of *The Journal of Headache and Pain* depends on the qualified and regular collaboration of renowned scientists, who devoted their time to constructively review the submitted articles.

We are indebted to the following experts who reviewed papers that completed the peer-reviewing process within 2014.

**M.A. Al-Jumah**

(Riyadh)

**A.P. Andreou**

(London)

**A. Antal**

(Göttingen)

**F. Antonaci**

(Pavia)

**R. Arcioni**

(Rome)

**A. Ashkenazi**

(Doylestown)

**M. Ashina**

(Copenhagen)

**S. Ashina**

(New York)

**T. Bartsch**

(Kiel)

**E. Beghi**

(Milano)

**A.C. Belin**

(Stockholm)

**L. Bendtsen**

(Copenhagen)

**R. Benoliel**

(Jerusalem)

**L. Berliocchi**

(Catanzaro)

**M. Bjørn Russell**

(Oslo)

**A. Bozzao**

(Rome)

**F. Brighina**

(Palermo)

**O. Bruni**

(Rome)

**J.M. Bugnicourt**

(Amiens)

**R. Cady**

(Springfield)

**L. Cao**

(Biddeford)

**S. Ceruti**

(Milan)

**S. Cevoli**

(Bologna)

**A. Charles**

(Los Angeles)

**T.C. Chaves**

(Ribeirão Preto)

**G. Coloprisco**

(Rome)

**G. Coppola**

(Latina)

**S. Cortese**

(Verona)

**C. Costa**

(Perugia)

**M. Davis**

(Rochester)

**M. de Tommaso**

(Bari)

**C. Di Lorenzo**

(Latina)

**V. Di Piero**

(Rome)

**H.C. Diener**

(Essen)

**A. Donnet**

(Paris)

**J. Ellrich**

(Aalborg)

**M. Engstrøm**

(Trondheim)

**D. Fanelli**

(Montreal)

**C. Fernández-de-Las-Peñas**

(Madrid)

**M. Fioravanti**

(Rome)

**J.L. Fuh**

(Taipei)

**F. Galli**

(Pavia)

**A.R. Gantenbein**

(Zurzach)

**C. Gaul**

(Essen)

**P. Gazerani**

(Aalborg)

**J. Genizi**

(Haifa)

**G. Gentile**

(Rome)

**P. Geppetti**

(Florence)

**P.J. Goadsby**

(San Francisco)

**F. Granella**

(Parma)

**K. Hagen**

(Trondheim)

**J.K. Hesselink**

(Utrecht)

**J. Hoffmann**

(Berlin)

**D. Holle**

(Essen)

**T.P. Jürgens**

(Hamburg)

**M.A. Kabbouche**

(Cincinnati)

**D.G.A. Kasteleijn-Nolst Trenite**

(Utrecht)

**Z. Katsarava**

(Unna)

**S. Khuder**

(Toledo)

**M. Koutris**

(Amsterdam)

**T. Jürgens**

(Hamburg)

**G. Lambru**

(London)

**M. Lantéri-Minet**

(Nice)

**J.P. Lefaucheur**

(Créteil)

**V. Limmroth**

(Cologne)

**M. Linde**

(Trondheim)

**R. Lipton**

(New York)

**S. Maabjerg**

(Glostrup)

**F. Maggioni**

(Padua)

**E. Magiorkinis**

(Athens)

**D.L. Maier**

(London)

**N. Maleki**

(Boston)

**G.C. Manzoni**

(Parma)

**M. Marmura**

(Phildadelphia)

**M. Matharu**

(London)

**P. Matusz**

(Lausanne)

**A. May**

(Hamburg)

**J. McDougall**

(Santa Rosa)

**A. Milde-Busch**

(Munich)

**M. Monticone**

(Pavia)

**S. Nelson**

(Boston)

**A. Negro**

(Rome)

**A. Özge**

(Yenişehir)

**D.S. Paauw**

(Seattle)

**K. Paemeleire**

(Ghent)

**A. Paglialonga**

(Milan)

**S.L. Pan**

(Taipei)

**J.A. Pareja**

(Alcorcón)

**P. Parisi**

(Rome)

**J. Pascual**

(Santander)

**J. Passchier**

(Amsterdam)

**D.B. Pfau**

(Mannheim)

**S. Prakash**

(Baroda)

**A. Raggi**

(Milan)

**I. Rainero**

(Turin)

**M. Robbins**

(New York)

**M.B. Russell**

(Oslo)

**S. Sacco**

(L’Aquila)

**F. Sakai**

(Saitama)

**P. Sandor**

(Zurich)

**P. Sarchielli**

(Perugia)

**A. Scher**

(Bethesda)

**E. Schmutzhard**

(Innsbruck)

**M. Schürks**

(Essen))

**G. Serafini**

(Rome)

**H. Shehata**

(Cairo)

**M. Sillanpää**

(Turku)

**A. Signore**

(Roma)

**M.M.S. Sindou**

(Lyon)

**N.N. Singh**

(Columbia)

**A. Siva**

(Istanbul)

**J. Smith**

(Lexington)

**A. Sonawalla**

(Karachi)

**A. Straube**

(Munich)

**O. Summ**

(Münster)

**T. Takeshima**

(Osaka)

**C. Tassorelli**

(Pavia)

**R. Teggi**

(Milan)

**P.C. Tfelt-Hansen**

(Glostrup)

**M. Thompson**

(Toronto)

**D. Toni**

(Rome)

**P. Torelli**

(Parma)

**A. Truini**

(Rome)

**B. Tsang**

(London)

**L. Valentinis**

(Portogruaro)

**M. Valeriani**

(Rome)

**A.M. Velly**

(Montreal)

**A. Verhagen**

(Rotterdam)

**G. Zanchin**

(Padua)

**D. Wagner**

(Glasgow)

**R. Walder**

(Iowa City)

**S.J. Wang**

(Taipei)

**X. Wang**

(Bermont)

**C. Wöber**

(Vienna)

**C. Wöber-Bingöl**

(Vienna)

**S.Y. Yu**

(Beijing)

**J.M. Zakrzewska**

(London)

